# The genetic etiology of hearing loss in Japan revealed by the social health insurance-based genetic testing of 10K patients

**DOI:** 10.1007/s00439-021-02371-3

**Published:** 2021-10-01

**Authors:** Shin-ichi Usami, Shin-ya Nishio

**Affiliations:** Department of Hearing Implant Sciences, Shinshu University School of Medicine, 3-1-1 Asahi, Matsumoto, 390-8621 Japan

## Abstract

Etiological studies have shown genetic disorders to be a major cause of sensorineural hearing loss, but there are a limited number of comprehensive etiological reports based on genetic analysis. In the present study, the same platform using a diagnostic DNA panel carrying 63 deafness genes and the same filtering algorithm were applied to 10,047 samples obtained from social health insurance-based genetic testing of hearing loss. The most remarkable result obtained in this comprehensive study was that the data first clarified the genetic epidemiology from congenital/early-onset deafness to late-onset hearing loss. The overall diagnostic rate was 38.8%, with the rate differing for each age group; 48.6% for the congenital/early-onset group (~5y.o.), 33.5% for the juvenile/young adult-onset group, and 18.0% for the 40+ y.o. group. Interestingly, each group showed a different kind of causative gene. With regard to the mutational spectra, there are certain recurrent variants that may be due to founder effects or hot spots. A series of haplotype studies have shown many recurrent variants are due to founder effects, which is compatible with human migration. It should be noted that, regardless of differences in the mutational spectrum, the clinical characteristics caused by particular genes can be considered universal. This comprehensive review clarified the detailed clinical characteristics (onset age, severity, progressiveness, etc.) of hearing loss caused by each gene, and will provide useful information for future clinical application, including genetic counseling and selection of appropriate interventions.

**Supplementary Information:**

The online version contains supplementary material available at 10.1007/s00439-021-02371-3.

## Introduction

Hearing loss is an extremely heterogenous disorder, and more than 120 genes are currently considered to be implicated in non-syndromic hearing loss, making the screening strategy difficult. Targeted genome resequencing using massively parallel DNA sequencing (MPS) has become a powerful strategy for the identification of causative genes from among the large numbers of genes in rare Mendelian disorders such as deafness. This sequencing technology followed by an appropriate filtering algorithm will be able to identify rare responsible genes for individual hearing loss patients. Although genetic disorders are thought to be a major cause of sensorineural hearing loss, there are a limited number of comprehensive etiological reports based on genetic analysis. In addition, it is difficult to draw conclusions without using the same analysis platform, filtering method, and pathogenicity assessment for samples collected by the same criteria. Through collaborative study with 102 collaborative centers in Japan, over 10,000 samples obtained from social health insurance-based testing and detailed clinical data have been collected. In this paper, we not only reviewed our series of studies based on analysis by MPS conducted over the last decade, but also performed comprehensive verification using the same analytic method and criteria.

## Genetic epidemiology based on genetic testing

Etiological studies have shown that genetic causes are the most common etiology of deafness, and approximately two-thirds of congenital/early-onset sensorineural hearing loss in developed countries is estimated to be due to genetic causes (Morton and Nance 2006). Recent studies have indicated that a significant portion of late-onset hearing loss is also due to genetic causes (Kitano et al. 2017; Kobayashi et al. 2018; Shinagawa et al. 2020a; Yasukawa et al. 2019; Oka et al. 2020; Miyajima et al. 2020). A series of etiological studies has demonstrated genetic disorders to be a common cause of all types of sensorineural hearing loss, but there has been no detailed genetic epidemiological data covering a wide range of ages.

Hereditary hearing impairment is an extremely heterogenous disorder that involves more than 120 distinct genes, thereby making the precise diagnosis and appropriate intervention difficult. Recent advances in targeted genome resequencing using massively parallel DNA sequencing (MPS) has provided a powerful new strategy and revolutionized the elucidation of genetic defects causing monogenic disorders. We have shown that this approach is appropriate for identifying causative genes/variants and actually demonstrated that various genes/gene variants are involved in hearing loss in Japanese patients (Miyagawa et al. 2013; Nishio et al. 2015). Meanwhile, both the number of DNA samples and detailed clinical data are increasing, and genetic and clinical data from over 10,000 patients has been collected from 102 collaborative centers. Based on this large-cohort data, we have published a series of studies demonstrating the mutational spectrum and clinical features caused by the representative deafness genes, including *GJB2* (Tsukada et al. 2010), *CDH23* (Miyagawa et al. 2012), *KCNQ4* (Naito et al. 2013), *OTOF* (Iwasa et al. 2013, 2019), mitochondrial 1555A > G and 3243A > G (Yano et al. 2014), *SLC26A4* (Miyagawa et al. 2014), *LRTOMT* (Ichinose et al. 2015), *GRXCR1* (Mori et al. 2015a), *PTPRQ* (Sakuma et al. 2015), *COCH* (Tsukada et al. 2015a), *TMPRSS3* (Miyagawa et al. 2015d), *STRC* (Moteki et al. 2016; Yokota et al. 2019), *LOXHD1* (Mori et al. 2015b; Maekawa et al. 2019), *ACTG1* (Miyagawa et al. 2015b; Miyajima et al. 2020), *MYO15A* (Miyagawa et al. 2015a), *POU4F3* (Kitano et al. 2017), *WFS1* (Kobayashi et al. 2018), *CLDN14* (Kitano et al. 2019), *EYA4* (Shinagawa et al. 2020a, b), *TECTA* (Yasukawa et al. 2019), *OTOA* (Sugiyama et al. 2019), and *MYO6* (Miyagawa et al. 2015c; Oka et al., 2020). These studies were performed between 2010 and 2020. It seems that the reported clinical characteristics of hearing loss caused by each gene do not significantly differ from the contents of our original papers, but the present review seeks to summarize the findings as more accurate and comprehensive results can now be obtained for the following reasons; (1) the samples and clinical data from hearing loss patients are increasing (approximately 700 samples per year), (2) the public database for normal controls is being updated, (3) disease-specific databases can be referenced, and (4) the results for the same hearing loss population using the same filtering algorithm can be compared.

## Unbiased samples obtained from social health insurance-based testing

In Japan, genetic testing for deafness, which has been reimbursed by the social health insurance system since 2012, has become a standard diagnostic tool for deafness. Collecting DNA samples for research purposes inevitably results in biased samples, but the biggest advantage of the social health insurance-based testing is that it is accessible to everyone and, therefore, more unbiased samples can be collected. This is important when discussing etiology.

Currently, DNA samples as well as clinical data from 10,047 patients have been collected from 102 collaborative centers participating in the deafness consortium, and the relationships between causative gene variants and clinical features have become clear. This review summarizes the findings, including mutational spectra and genotype/phenotype correlations, obtained from the large-cohort data.

The samples used in this review were as follows. With regard to inheritance mode, 2243 subjects were from autosomal dominant or mitochondrial inherited families, 6163 subjects from autosomal recessive families or sporadic cases, and 1641 showed unknown inheritance mode. Patients for whom the onset age (the age of awareness) was available numbered 3877 prelingual hearing loss (below age 6), 2698 post-lingual hearing loss (aged between 6 and 39), and 1057 late-onset hearing loss (after the age of 40) cases. Hearing levels were classified based on the better hearing ear as normal, < 20 dB; mild hearing loss, 21–40 dB (*n* = 1162); moderate hearing loss, 41–70 dB (*n* = 2746); severe hearing loss, 71–95 dB (*n* = 1622); and profound hearing loss, > 95 dB (*n* = 1660). All subjects had presumed non-syndromic sensorineural hearing loss (SNHL). The samples were obtained primarily from bilateral SNHL, but also contained 220 samples from unilateral SNHL patients. Written informed consent was obtained from all patients (or from their next of kin, caretaker or guardian in case of minors or children), and the study was approved by the Shinshu University Ethical Committee as well as the relevant bodies of the other participating institutions of the Deafness Gene Study Consortium. Clinical information and peripheral blood samples were obtained from each subject and from all their consenting relatives. This study was conducted in accordance with the Declaration of Helsinki, and the protocol was approved by the Ethics Committee of Shinshu University School of Medicine (No. 387 ~ 4 September 2012, No. 576 ~ 2 May 2017).

## Sequencing strategy and filtering algorithm

Sixty-three genes (shown in Supplementary Table S1; Ion AmpliSeq™ Hearing Loss Research Panel v1, Thermo Fisher Scientific, Waltham, MA USA), reported to be causative of non-syndromic hearing loss (Hereditary Hearing loss Homepage; http://hereditaryhearingloss.org/), were analyzed in this study. The detailed protocols for targeted genome enrichment and massively parallel DNA sequencing have been described elsewhere (Nishio et al. 2015). In addition to MPS, copy number variation (CNV) analysis was performed using the same platform as for MPS in accordance with our previous report (Nishio et al. 2018).

For the filtering algorithm used in the present MPS analysis, (1) allele frequency in a public database, (2) a disease-specific database, (3) variant type, and (4) in silico prediction score were used. For clinical setting and practical genetic diagnosis, a great deal of the above-mentioned information needs to be integrated and evaluated. To integrate (1)–(4), we have developed an integrated database of clinical information and genetic information from patients with genetic deafness (Nishio and Usami 2017). It should be emphasized that this database is particularly powerful for selecting candidate gene variants, and we have already collected detailed clinical information and genetic analysis data from more than 10,000 hearing loss patients. In the case of rare diseases such as hereditary hearing loss, it is often difficult to determine whether the variant found in a specific patient is pathogenic. However, the pathogenicity can be determined efficiently using a database containing information obtained from large number of hearing loss patients.

The pathogenicity of the identified variants was evaluated in accordance with the American College of Medical Genetics (ACMG) standards and guidelines (Richards et al. 2015) with the ClinGen hearing loss clinical domain working group expert specification (Oza et al. 2018). Variants were defined as likely causative variants if the following criteria was fulfilled; (1) for the variants previously reported as “pathogenic” or “likely pathogenic” and did not show any contradictory evidence, we applied the same pathogenicity classification as the previous report (HGMD professional), with reference to the pathogenicity classification in the Deafness variation database ver. 9 (Azaiez et al. 2018) and ClinVar database ver. 20210501 (Landrum et al. 2018), (2) novel variants classified as “pathogenic” or “likely pathogenic” were considered as strong candidates for each case, (3) variants of “uncertain significance” (VUS) identified as the only candidate after the filtering procedure with no other candidate variants in the other 62 genes were also included, (4) two variants found in recessive inheritance cases, (5) there was no contradiction with the results of family analysis, and (6) in cases with conflicting findings between our pathogenicity classification and the HGMD professional, Deafness variation database and ClinVar database, we manually curated the data based on the ACMG guidelines with reference to the patient’s clinical information.

## Responsible genes in the Japanese hearing loss population

**Fig. 1:**
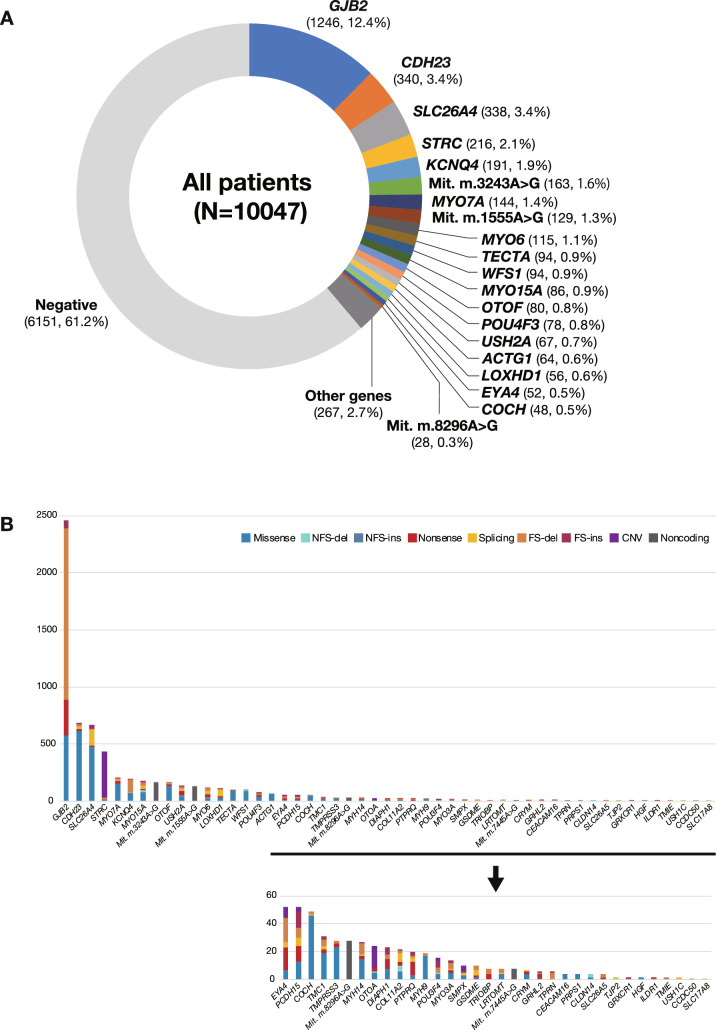
**A** Responsible genes found in 10,047 hearing loss patients. **B** The number and types of variants in each gene

Using simultaneous screening for 63 deafness genes, we identified 51 genes that are definitely involved in the Japanese hearing loss population (Table S2). Further, using the criteria mentioned above, the overall diagnostic rate was 38.8% (3896/10,047); i.e., one causative variant in autosomal dominant or mitochondrial inheritance cases, and biallelic causative variants in recessive inheritance or sporadic cases (Fig. 1A).

To determine which genes among those identified have the greatest impact on the etiology of deafness, the number of variants and variant types (missense, nonsense, frameshift) were calculated. Results showed that the number of mutations in *GJB2* was exceptionally high, followed by mutations in *CDH23, SLC26A4, STRC, KCNQ4,* mitochondrial m. 3243A > G*, MYO7A,* mitochondrial m.1555A > G*, MYO6, TECTA, WFS1, MYO15A, OTOF, POU4F3* and *USH2A*. (Fig. 1A, B). *GJB2* is the most prevalent causative gene, with several major (commonly found) gene mutations causing deafness in 30–35% of cases, while the remaining cases of hearing loss are the result of various rare genes/mutations that have been efficiently diagnosed by the present sequencing and CNV detection approach (Fig. 1A, B). The high prevalence of *GJB2* mutations in the hearing loss population is evident regardless of ethnic differences. It is hypothesized that having variants in *GJB2* was beneficial for survival. *GJB2* is also known to be expressed on the skin, and the skin of subjects with *GJB2* mutations is thicker (the skin is a barrier against pathogen invasion, trauma, and insect bites), which was beneficial to survival associated with human evolution (Meyer et al. 2002). This hypothesis could be supported from the prevalence of LOF (loss of function) mutations observed in the Genome Aggregation Database (gnomAD, https://gnomad.broadinstitute.org). Based on the gnomAD, in contrast to the other deafness genes where the number of “observed” LOFs (loss of function variants) is equal to that of “expected” LOFs, the number of “observed” LOFs (*n* = 17) in *GJB2* is exceptionally high compared to that of “expected” LOFs (*n* = 6.5) (see *GJB2* on the gnomAD home page), indicating that there was a certain benefit in having *GJB2* mutations in ancient times.

Table 1 shows a summary of the previous papers describing the results of genetic screening using MPS. It is a consensus fact that variants in *GJB2* are predominant across all ethnic groups, but there are also some exceptions as shown in Table 1. For example, *MYO15A* and *TMC1* are predominant in consanguineous Egyptian families and Sephardi Jews, respectively (Brownstein et al. 2020; Budde et al. 2020). This specific causative gene rate is probably the result of consanguineous marriage, which is common in these regions.

**Table 1:** Previous papers describing the results of genetic screening using MPS

Author (year)	Target genes	Mutated genes	Subjects	Ethnicity/nationality	Methods	Diagnostic rate	Most frequent	2nd frequent	3rd frequent	4th frequent	5th frequent
Miyagawa et al. (2013)	112	57	216 (120 early-onset/96 late-onset)	Japanese	MPS	NA (86.6% 187/216 variant detection rate)	*GJB2*	*SLC26A4*	*USH2A*	*GPR98*	*MYO15A*
Yang et al. (2013)	79	23	190 (early-onset/late-onset)	Chinese Hans	MPS	51.6% (98/190)	*GJB2*	*SLC26A4*	*m.1555A* > *G*	*MYO15A*	*USH2A, PCDH15, GPR98, TMC1*
Nishio and Usami (2015)	63	NA	1120 (early-onset/late-onset)	Japanese	MPS	NA	*GJB2*	*CDH23*	*SLC26A4*	*MYO15A*	*COL11A2*
Sloan-Heggen et al. (2016)	66/89	49	1119 (congenital/childhood/adulthood)	Various	MPS + CNV	39.3% (440/1119)	*GJB2*	*STRC*	*SLC26A4*	*TECTA*	*MYO15A*
Sommen et al. (2016)	79	16	131 (GBJ2 excluded/ARNSHL/prelingual moderate-profound)	Western European	MPS + CNV	23.7% (31/131)	*GJB2* (excluded)	*TMC1, MYO7A, MYO15A*			
Yan et al. (2016)	180	27	342 (GJB2 excluded)	Various (91 indigenous families from South Africa, 90 from Nigeria, 53 from the USA (South Florida), 38 from Tunisia, 23 from India, 21 from Iran, 19 from Turkey, and 7 from Guatemala)	MPS	15% (53/342)	*GJB2* (excluded)	*MYO15A*	*SLC26A4*	*USH2A*	*MYO7A*
Bademci et al. (2016)	WES	31	160 (GJB2 excluded/ARNSHL)	Mostly Turkey	WES	56% (90/160)	*GJB2* (excluded)	*MYO15A*	*MYO7A*	*SLC26A4*	*TMPRSS3*
Seco et al. (2017)	120	27	200 (79 congenital/60 first-decade onset)	Netherland	MPS + CNV	33.5% (67/200)	*GJB2*	*USH2A*	*MYO15A*	*STRC*	*CDH23*
Baux et al. (2017)	74	19	207 (NSHL)	French	MPS + CNV	48% (85/207)	*GJB2*	*STRC*	*MYO7A*	*MYO15A, TECTA, USH2A*	
Morgan et al. (2018)	96	20	103 (GJB2 excluded)	Italian	MPS + CNV	31% (32/103)	*GJB2* (excluded)	*TECTA*	*ACTG1, TMPRSS3*		*STRC*
Cabanillas et al. (2018)	199	16	50 (GJB2/GJB6, OTOF and MT-RNR1 excluded)	Spanish	MPS + CNV	42% (21/50)	*GJB2* (excluded)	*ACTG1*	*USH2A*	*STRC*	
Sun et al. (2019)	127	24	58	Chinese	MPS	77.59% (45/58)	*SLC26A4*	*GJB2*	*MYO15A*	*TRIOBP*	*MYO7A*
Wu et al. (2019)	80/213	27	280 (1291 of 5184 were excluded with GJB2, SLC26A4, m.1555A > G mutation)	Taiwanese	MPS	30.7% (86/280)	*GJB2* (excluded)	*SLC26A4* (excluded)	*OTOF*	*MYO15A*	m.1555A > G (excluded)
Abu Rayyan et al. (2020)	181	48	491 families	Palestinian	MPS	52% (254/491), 87% (201/231 multiplex famlies), 47% (111/227 singleton families)	*GJB2*	*SLC26A4*	*MYO15A*	*MYO7A*	*CDH23*
Yuan et al. (2020)	129	24	463 (433 sporadic SNHL/30 deafness pedigree)	Chinese	MPS	52.19% (226/433 sporadic SNHL), 56.67% (17/78 deafness pedigree)	*GJB2*	*SLC26A4*	m.1555A > G	*MYO15A*	*POU3F4, USH2A, MYO1F, MYO7A, TMC1*
García-García et al. (2020)	59	25	128 (118 families): 120 NS, 4USH, 2WS, 2BOR	Spanish	MPS + CNV	40% (47/118)	*GJB2*	*MYO6*	*TECTA*	*STRC*	*LOXHD1, OTOA, OTOF, COL11A2, WFS1*
Budde et al. (2020)	12/94	23	61 consanguineous families	Egyptian	MPS	79% (48/61)	*MYO15A*	*SLC26A4*	*GJB2*	*MYO7A*	*ADGRV1, BSND, CDH23, TECTA, TRIOBP, LOXHD1*
Morgan et al. (2020)	WES	27	125 (118 NSHL, 5 SHL)	Italian	WES + CNV	50% (58/118)	*GJB2*	*STRC*	*USH2A*	*MYO7A*	
Safka Brozkova et al. (2020)	41/84/WES	16	421 (early onset below the first few years)	Czech	CNV + MPS + WES	12.8% (54/421)	*GJB2* (excluded)	*STRC*	*LOXHD1, MYO15A, TMPRSS3*		
Brownstein et al. (2020)	178/372	35	88 (muliplex family)	Jewish (Ashkenazi, Mizrahi, Sephardi)	MPS	60% (53/88)	*GJB2*(Ashkenazi, Mizrahi), *TMC1* (Sephardi)	*MYO15A* (Ashkenazi, Mizrahi), *CDH23* (Sephardi)			
This study (2021)	63	51	10,047 [congenital (3877), 6–39 y.o.(2698), over 40 (1057)]	Japanese	MPS + CNV	38.8% (3899/10047), congenital: 48.6% (1886/3877), 6–39 y.o.: 33.5% (905/2698), over 40: 18.1% (191/1057)	*GJB2*	*CDH23*	*SLC26A4*	*STRC*	*KCNQ4*

*WES* whole exome sequencing, *MPS* massively parallel DNA sequencing, *CNV* copy number variation, *AR* autosomal recessive, *NSHL* non-syndromic hearing loss, *SHL* syndromic hearing loss, *USH* Usher syndrome, *WS* Waardenburg syndrome

Several causative genes, such as *SLC26A4, MYO15A, MYO7A,* and *CDH23*, are frequently found across different populations, although there are some ethnic differences in the second and third most frequent genes. These differences are most likely due to founder mutations occurring during human migration, as these ethnic differences are evident even in analyses using the same screening platform (Sloan-Heggen et al. 2016). However, it is important to keep in mind that such differences may also be caused by other factors, including sampling bias, method used, differences in filtering procedure, and differences in the definitions of pathogenicity. In connection with sampling bias and the methodology used, when CNV analysis is performed or when mild-to-moderate deafness is included, a certain number of patients with hearing loss caused by CNV (copy loss) of the *STRC* gene could be identified (Sloan-Heggen et al. 2016; this study).

Regardless of the number of genes in the diagnostic panel (number ranges from 12 to 327 or the whole exome), the number of mutated genes is somewhat limited (maximum: 57). These results converge to around 50–60 genes, even when many genes are analyzed. There probably are, however, many extremely rare genes in the various hearing loss populations.

The present comprehensive study revealed certain tendencies in terms of variant type. In general, gene mutations in AR deafness tend to have more LOF mutations; *GJB2* (frequency of LOF: 76.7%), *STRC* (97.2%), *MYO15A* (44.8%), *LOXHD1* (71.4%), *USH2A* (64.9%), and *PCDH15* (75.0%). However, even in AR genes, if the founder mutation is a missense mutation, the number of LOF mutations is slightly lower; for example, *SLC26A4* (p. His723Arg, a known founder mutation, 28.1%) and *OTOF* (p.Arg1939Gln a known founder mutation, 18.5%). Also, *CDH23* has a very low LOF percentage (9.5%), probably as mutations lead to Usher syndrome. In the case of the gene mutations in AD deafness, there are two patterns regarding variant type; (1) genes that have many missense and a low frequency of LOF mutations, such as *TECTA* (7.1%), *WFS1* (0%), *ACTG1* (0%), *COCH* (6.1%), and *MYH9* (10.5%), and (2) genes that have a high frequency of LOF and few missense mutations, such as *KCNQ4* (62.3%), *MYO6* (75.9%), *POU4F3* (51.3%), and *EYA4* (86.3%). The mechanisms by which the mutations cause deafness are thought to differ; a dominant negative mechanism for the former and haploinsufficiency for the latter group.

**Fig. 2:**
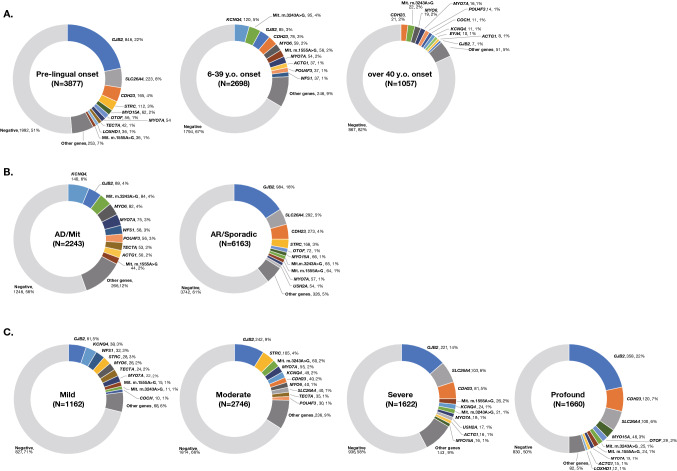
**A** Responsible genes in each age group (congenital/6–39 y.o./over 40 y.o.). **B** Responsible genes found in autosomal dominant (AD) and autosomal recessive (AR) patients. Among 89 *GJB2*-associated hearing loss cases identified from autosomal dominant families, 23 carried autosomal dominant inheritance variants and 66 carried biallelic autosomal recessive variants (pseudo-dominant cases). Similarly, among 75 *MYO7A*-associated hearing loss cases identified from autosomal dominant families, 71 carried autosomal dominant inheritance variants and only 4 carried biallelic autosomal recessive variants (pseudo-dominant cases). **C** Responsible genes found in groups classified by severity of HL. It was revealed that the types of genes differed according to severity

**Fig. 3:**
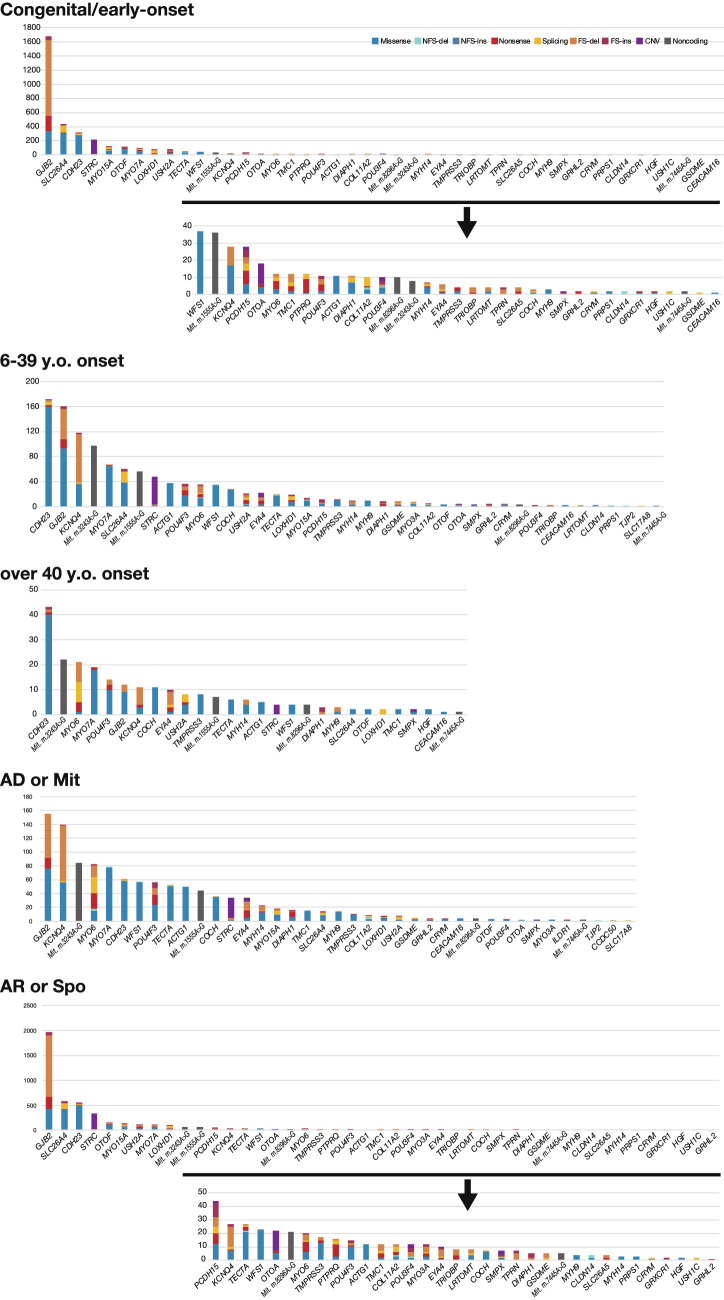
The number and types of variants in each gene identified in each age group (congenital/6–39 y.o./over 40 y.o.) and in autosomal recessive (AR) and autosomal dominant (AD) patients

Samples collected in a more unbiased manner from social health insurance-based testing cover a wide range of age groups and reveal the comprehensive genetic epidemiology in Japanese. With regard to age at onset, we divided the samples into 3 groups by age; congenital/early-onset (~ 5 y.o.), juvenile/young adult-onset (6–39 y.o.), and middle aged/aged-onset (40 y.o ~). The diagnostic rate was higher in the congenital/early-onset group (~ 5 y.o.) (48.6%; 1885/3877) (Fig. 2A), with variants in *GJB2*, *SLC26A4, CDH23, STRC,* and *MYO15A* frequently observed. The diagnostic rate in the juvenile/young adult-onset group was lower than that in the congenital/early-onset group, but the responsible genes were detected in a significant portion of the patients (Fig. 2A) (33.5%; 904/2698). It is well known that many different genes are involved in congenital/early-onset hearing loss, and the present study revealed that many genes are also involved in late-onset hearing loss. Interestingly, the responsible genes differed from those found in the congenital/early-onset group. The present analysis revealed that genes such as *KCNQ4,* mitochondrial mutations (m.1555A > G and m.3243A > G), *GJB2, CDH23, MYO6* and *MYO7A* were frequently found in the juvenile/young adult-onset group. The causative gene was found in only a few patients in the over 40 y.o. group (18.0%; 190/1057) (Figs. 2A, 3) and genetic involvement was relatively low in the subjects over 40 years, suggesting that other factors such as environmental factors (exposure to noise, aging, etc.), or other unknown genes (either Mendelian or multifactorial inheritance) may be involved. However, it is still interesting to note that the characteristic genes in this group were *CDH23,* mitochondrial m.3243A > G*, MYO6, MYO7A, POU4F3* and *COCH*. *GJB2* and *CDH23* were found in all groups, but it should be noted that the identified mutations differed; i.e., p.Val37Ile in *GJB2,* and p.Arg1588Trp and p.Arg2029Trp in *CDH23* were predominantly found in the oldest group. As p.Val37Ile, the causative variant of the milder phenotype as shown in our previous study (Tsukada et al. 2010), was found in older patients, it probably reflects the age at diagnosis by school and workplace annual health examinations, rather than the age at onset. Actually, in this study, the proportion of p.Val37Ile patients among those with *GJB2* mutations was 10.2% (86/846) in the congenital group, compared to 52.9% (45/85) in the juvenile and young adult-onset (6–39 y.o.) group, supporting our previous results.

*GJB2, SLC26A4, CDH23, STRC, OTOF* and *MYO15A* were commonly found to be the responsible genes in autosomal recessive/sporadic inheritance cases, and *KCNQ4*, mitochondrial m.3243A > G, *MYO6*, *MYO7A, WFS1, POU4F3* and *TECTA* were frequently identified in autosomal dominant inheritance cases (Figs. 2B, 3).

It was also revealed that the types of genes differed in the groups according to severity; i.e., *GJB2, SLC26A4,* and *CDH23* were more commonly found in the severe-to-profound hearing loss group, while *GJB2, STRC, KCNQ4, WFS1, MYO7A,* and *MYO6* were found to be more common in the mild-to-moderate hearing loss group (Fig. 2C).

The fact that the causative gene was only found in one case (mitochondrial m.1555A > G) out of the 220 unilateral hearing loss cases suggested that factors other than genetic factors were involved in unilateral hearing loss.

## Recurrent variants: founder effect or mutational hot spot?

The identified variants are shown in Supplemental Table S2, which shows that our series of studies revealed certain recurrent mutations, which is crucial for molecular diagnosis to allow decision-making with regard to the appropriate intervention (Ohtsuka et al. 2003; Miyagawa et al. 2014). Based on such recurrent mutations in the Japanese population, we have developed an Invader screening assay that has been proven to give satisfactory results (Abe et al. 2007; Usami et al, 2012b). It is generally accepted that recurrent genetic mutations occur via two mechanisms: one is a founder effect and the other is a mutational hot spot, and haplotype analysis was performed to reveal whether these mutations occur by a founder effect or mutational hot spot.

**Fig. 4:**
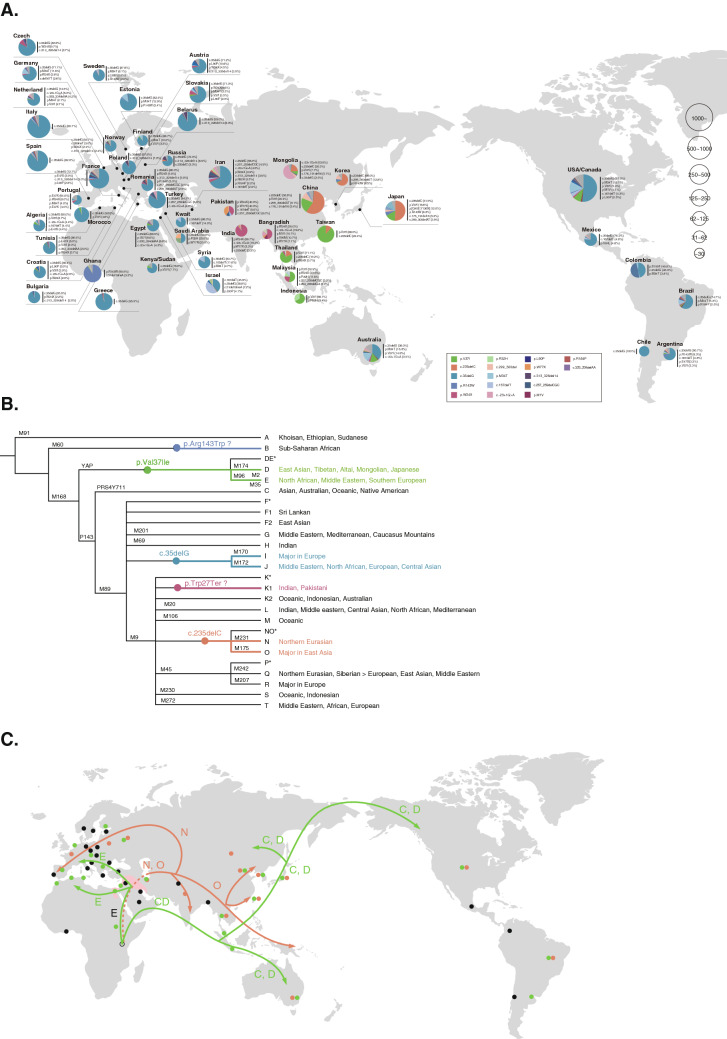
A The spectrum of *GJB2* mutations. A larger circle indicates a larger number of mutated alleles (from Tsukada et al. 2015b). **B** Suspected origin of the *GJB2* variants (modified from Tsukada et al. 2015b)**.** Suspected origin of *GJB2* variants are marked on the human Y-chromosomal haplogroup tree (Karafet et al. 2008), which is applicable to the investigation of human migration. Lineages associated with haplogroup IJ in the Y-haplotype tree based on Karafet et al. (2008). From geographical distribution and haplotype analysis, it is speculated that p. Arg143Trp may have occurred in the Y-chromosomal haplogroup B, p.Val37Ile in D and E, and c.235delC, p.[G45E; Y136X], and c.176_191del, c.299_300delAT in N and O, respectively. **C** *GJB2* founder variants from a human migration perspective based on Y chromosome haplotypes: c.235 del C is considered to have occurred in haplotype N/O from the viewpoint of its geographic distribution. On the other hand, p.Val37Ile is consistent with the area where the haplotype C/D/E was found, so it is probable that it occurred within this haplotype

The most readily understandable example is *GJB2*, the most common deafness gene. Numerous pathogenic variants have been demonstrated over the past two decades and the reported mutational spectra are known to be ethnicity specific. There are great variations in the allele frequency of patients with *GJB2* mutations in each population, suggesting that the allele frequency in the population, which reflects a founder effect, strongly affects the status of the *GJB2* gene in the hearing loss population (Tsukada et al. 2015b). As certain mutations are clearly found in only limited ethnic populations, it is possible to predict that those mutations are due to a founder effect (Fig. 4A).

We have reviewed the reported *GJB2* variants in different populations and considered them from the perspective of human migration (Tsukada et al. 2015b). Founder effects have received special emphasis from this perspective. The c.35delG variant, which is predominant throughout Europe, the Middle East, North Africa, North and South America, and Australia, has been proven by haplotype analysis to be due to a founder effect occurring 11,000 years ago (van Laer et al. 2001). With regard to six frequent *GJB2* variants observed in Japanese hearing loss patients (i.e., c.235delC, p.Val37Ile, p.[Gly45Glu; Tyr136Ter], p.Arg143Trp, c.176_191del, and c.299_300delAT), we concluded that the six variants were derived from founder effects as they were observed in a specific haplotype. The c.235delC variant, most frequently found in Japanese hearing loss population, was observed in countries in East and Central Asia, such as Japan, Korean, China, Mongolia, and Thailand, whereas it is rarely observed in other populations. Such an uneven distribution of this variant suggests that it was caused by a founder effect. The c.235delC variant is estimated to have first occurred at around 6500 years ago (Shinagawa et al. 2020b). Our haplotype analysis, together with their distribution patterns, indicated that the p.Arg143Trp and p.Val37Ile variants may have occurred as multiple events, suggesting that both a founder effect and hot spot may be involved in these variants. With regard to the founder age of frequent *GJB2* variants, each variant may have occurred at a different time, with the oldest, p.Val37Ile, considered to have occurred around 14,500 years ago, and the most recent, c.176_191del, considered to have occurred around 4000 years ago (Shinagawa et al. 2020b).

A comparative analysis of the results of clustering analysis and the phylogenetic tree based on the results of Y-chromosomal haplogroup analysis indicates that many variant distributions are well explained by founder effects in ancient human lineages (Fig. 4B, C). The p.Arg143Trp and p.Val37Ile variants are spread widely across the globe and are speculated to have occurred at a very early stage in human migration. As these two mutations are frequently found in Japanese, as stated above, it is considered that the two mutations occurred multiple times. Our haplotype analysis indicated that p. Arg143Trp and p.Val37Ile are thought to have occurred in the Y-chromosomal haplogroup N as well as O (in the same haplotype as c.235delC, p. [Gly45Glu; Tyr136Ter], c.176_191del, and c.299_300delAT).

To date, various other genes have been extensively studied in a number of ethnic groups and certain recurrent mutations have been confirmed to be due to founder effects, such as *CDH23* (Kim et al. 2015), *MYO15A* (Palombo et al. 2017), and *TMC1* (Ramzan et al. 2020). Several founder mutations have also been reported in Japanese patients with hearing loss. A series of haplotype analyses indicated that p. His723Arg in *SLC26A4* (Park et al. 2003), p.Arg1939Gln in *OTOF* (Matsunaga et al. 2012), c.211delC in *KCNQ4* (Naito et al. 2013), and c.4212 + 1G > A in *LOXHD1* (Maekawa et al. 2019) are suspected to be founder variants. In contrast, c.5597C > T in *TECTA* (Yasukawa et al. 2019) and p.Ala716Thr, p.Lys836Thr, and p.Glu864Lys in *WFS1* (Kobayashi et al. 2018) occurred in mutational hot spots. As mentioned above, p.Val37Ile and p.Arg143Trp in *GJB2* are due to both a founder effect and a hot spot.

## Clinical characteristics

Regarding hereditary hearing loss, it is known that age at onset, severity, audiogram configuration, progressiveness, and presence of associated symptoms differ depending on the type of causative gene and variant, and site on the variant. We have collected data from a large number of Japanese hearing loss patients and clarified the clinical characteristics and genotype/phenotype correlation for each causative gene.

**Fig. 5:**
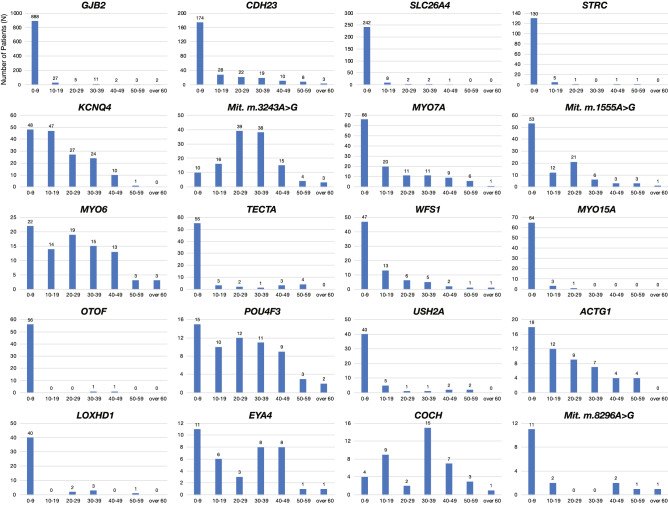
Age at onset (awareness) of each gene. Hearing loss related to *GJB2, CDH23, SLC26A4, STRC, TECTA, MYO15A, OTOF, USH2A,* and *LOXHD1* is shown to be congenital/early-onset. In contrast, a significant portion of cases with mutations in *KCNQ4,* mitochondrial m.3243A > G, *MYO6, POU4F3, ACTG1, EYA4,* and *COCH* showed adult-onset hearing loss

As to the onset age, Fig. 5 clearly shows that hearing loss related to *GJB2, CDH23, SLC26A4, STRC, TECTA, MYO15A, OTOF, USH2A,* and *LOXHD1* is congenital/early-onset. In contrast, a significant proportion of cases related to *KCNQ4*, mitochondrial m.3243A > G, *MYO6*, *POU4F*3, *ACTG1*, *EYA4* and *COCH* are adult-onset.

Regarding severity and audiogram configuration, it has been clarified that there is a genotype/phenotype correlation for *GJB2*, which is the gene most frequently observed in congenital hearing loss (Snoeckx et al. 2005; Tsukada et al. 2010). Also, particular genes such as *TECTA* and *WFS1* are associated with characteristic audiogram configurations that show mid-frequency- and low-frequency-involved hearing loss, respectively (Taylor et al. 2013). Such characteristic audiogram configurations have also been found to be related to the mutated domain; i.e., if the mutation is located in the ZP domain of the *TECTA* gene, a dish-shaped audiogram configuration is observed (Yasukawa et al. 2019). With regard to the causative gene of mild-to-moderate hearing loss, as mentioned above, the *GJB2* gene has been known to have genotype/phenotype correlations, and p.V37I variant-associated hearing loss is mild-to-moderate (Tsukada et al. 2015b). Recent CNV analyses have shed light on the etiology of this group. CNV analysis revealed the CNV of the *STRC* gene is the second most common etiology in cases of mild-to-moderate hearing loss after the *GJB2* gene (Yokota et al. 2019). We have developed an efficient way to identify CNV using the same MPS platform used for the social health insurance-based genetic testing (Nishio et al. 2018). In addition, the *OTOA* gene, which encodes otoancorin and has a role in anchoring the extracellular matrix of the tectorial membrane to the edge of the spiral plate, has also been clarified as the causative gene of mild-to-moderate deafness (Sugiyama et al. 2019).

**Fig. 6:**
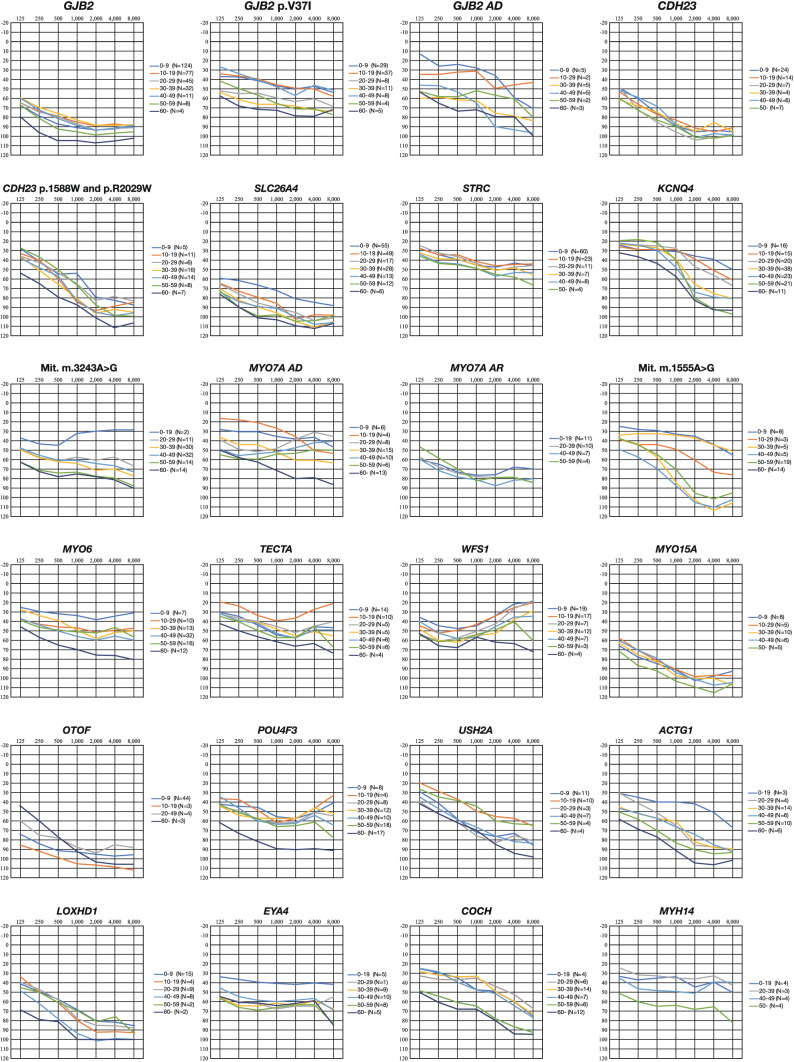
The average audiogram configuration and progressiveness. Hearing in each age group was plotted on the audiogram for each gene. In the case of the *GJB2* and *STRC* genes, hearing is rather stable. On the other hand, hearing loss due to *SLC26A4*, *CDH23*, *TMPRSS3*, *LOXHD1*, *KCNQ4*, *ACTG1*, *POU4F3*, *EYA4*, *MYO6*, and mitochondrial m.1555A > G showed progression with age

With regard to progression, in the case of the *GJB2* and *STRC*, hearing is rather stable and progression of hearing loss is rarely observed (Tsukada et al. 2010; Yokota et al. 2019). On the other hand, hearing loss due to *SLC26A4* (Suzuki et al. 2007; Miyagawa et al. 2014), *CDH23* (Miyagawa et al. 2012), *TMPRSS3* (Miyagawa et al. 2015c), *LOXHD1* (Maekawa et al. 2019), *KCNQ4* (Naito et al. 2013), *ACTG1* (Miyajima et al. 2020), *POU4F3* (Kitano et al. 2017), *EYA4* (Shinagawa et al. 2020a), *MYO6* (Oka et al. 2020), and mitochondrial m.1555A > G (Usami et al. 1997) requires a good deal of attention as it is progressive. Figure 6 demonstrates the progressive or non-progressive nature of each gene, and supports our previous data regarding progressiveness. From a clinical perspective, the most important information revealed by the analysis using the large-cohort data is whether the deafness is progressive and, if so, the progression rate. As deafness is a rare disease, it is difficult to analyze the progression of hearing loss from a small amount of data, with it only being possible from such large-cohort data as used in this study. This information is crucial for choosing an appropriate therapeutic intervention strategy, such as a hearing aid, electric acoustic stimulation (EAS) or conventional cochlear implantation (CI).

With regard to intervention, cochlear implantation is currently the standard therapeutic option for severe-to-profound hearing loss patients. The indications for CI are being expanded to patients with residual hearing and EAS, which uses both electric and acoustic stimulation at the same time, is indicated for such patients. Although CI provides a good outcome in the majority of cases, the outcomes still vary among patients. It is presumed that a number of factors are involved in such variability. Among them, genetic factors, which represent the most common etiology in severe-to-profound hearing loss, might be one of the key determinants of outcomes for CI and EAS (Usami et al. 2012a, 2020; Miyagawa et al. 2016). Causative mutations were successfully identified in 60% of patients with prelingual-onset hearing loss and in 36% of those with post-lingual hearing loss (Miyagawa et al. 2016). Based on our series of studies, good performance can be expected if the intra-cochlear etiology can be proven by gene identification (Miyagawa et al. 2016; Usami et al. 2020). Thus, determination of the involved regions inside/outside of the cochlea by identification of the responsible gene is crucial for selecting the appropriate intervention strategy.

In conclusion, although there are some ethnic differences in the mutational spectra, the genetic epidemiology and clinical features revealed by the present review study are believed to contain a general rule that transcends such ethnic differences.

## Supplementary Information

Below is the link to the electronic supplementary material.

Supplementary file1 (PDF 55 kb)

Supplementary file2 (XLSX 233 kb)
