# Supplementary material for: The genetic etiology of hearing loss in Japan revealed by the social health insurance-based genetic testing of 10K patients
Source: Hum Genet. 2021 Oct 1;141(3-4):665–81. doi: 10.1007/s00439-021-02371-3 (PMC9035015; doi:10.1007/s00439-021-02371-3)
Supplement: Supplementary file 1 — Supplementary file1 (PDF 55 kb) [file 439_2021_2371_MOESM1_ESM.pdf]

Supplementa Table 1. Genes including in Ion AmpliSeq™ Hearing Loss Research Panel v1

Gene Name	Chromosome	Num_Amplicons	Total_Bases	Covered_Bases	Overall_Coverage
ACTG1	chr17	11	1183	1183	1
CCDC50	chr3	20	1581	1572	0.9943
CDH23	chr10	108	12291	12289	0.99984
CEACAM16	chr19	14	1344	1335	0.9933
CLDN14	chr21	6	812	812	1
COCH	chr14	20	1774	1767	0.9961
COL11A2	chr6	73	6215	6203	0.9981
CRYM	chr16	11	1088	1088	1
DFNA5	chr7	16	1685	1681	0.9976
DIAPH1	chr5	40	4127	4107	0.9952
ESPN	chr1	26	2708	2511	0.9273
ESRRB	chr14	14	1615	1615	1
EYA4	chr6	32	2241	2235	0.9973
GIPC3	chr19	10	1005	835	0.8308
GJB2	chr13	5	714	714	1
GJB3	chr1	10	3228	3228	1
GJB6	chr13	6	812	812	1
GPSM2	chr1	29	2209	2148	0.9724
GRHL2	chr8	23	2054	2054	1
GRXCR1	chr4	9	917	917	1
HGF	chr7	32	2705	2685	0.9926
ILDR1	chr3	14	1729	1729	1
KCNQ4	chr1	21	2242	1974	0.8805
LHFPL5	chr6	7	693	693	1
LOXHD1	chr18	74	8125	8079	0.9943
LRTOMT	chr11	15	1644	1593	0.969
MARVELD2	chr5	15	1743	1687	0.9679
MSRB3	chr12	11	732	732	1
MYH14	chr19	66	6573	6570	0.9995
MYH9	chr22	65	6323	6318	0.9992
MYO15A	chr17	103	11297	10711	0.94813
MYO1A	chr12	34	3429	3425	0.9988
MYO3A	chr10	60	5214	5112	0.9804
MYO6	chr6	57	4232	3923	0.927
MYO7A	chr11	80	7652	7617	0.9954
OTOA	chr16	31	3788	2839	0.7495
OTOF	chr2	71	7115	7086	0.9959
PCDH15	chr10	86	8466	8365	0.9881
DFNB59	chr2	14	1125	1125	1
POU4F3	chr5	7	1039	1039	1
PTPRQ	chr12	83	6846	6445	0.9414
RDX	chr11	23	1895	1807	0.9536
SERPINB6	chr6	14	1197	1197	1
SLC17A8	chr12	18	1902	1873	0.9848
SLC26A4	chr7	31	2563	2482	0.9684
SLC26A5	chr7	25	2509	2432	0.9693
SMAC	chr12	7	786	786	1
STRC	chr15	25	5647	2010	0.3559
TECTA	chr11	54	6721	6698	0.9966
TJP2	chr9	43	4455	4253	0.9547
TMC1	chr9	30	2503	2386	0.9533
TMIE	chr3	6	515	515	1
TMPRSS3	chr21	14	1761	1684	0.9563
TPRN	chr9	13	2180	1582	0.7257
TRIOBP	chr22	59	7645	7273	0.9513
USH1C	chr11	38	3106	3106	1
WFS1	chr4	21	2750	2607	0.948
WHRN	chr9	25	3074	2998	0.9753
POU3F4	chrX	8	1097	1089	0.9927
PRPS1	chrX	11	1137	1137	1
SMPX	chrX	4	300	300	1
USH2A	chr1	164	16646	16567	0.99525
MIRN96	chr7	2	78	78	1
